# Lower Interhemispheric Coherence in Adults with Surgically Treated Severe Generalized Epilepsy than in Patients Without Epilepsy: A Scalp EEG Study

**DOI:** 10.3390/brainsci16020210

**Published:** 2026-02-11

**Authors:** Shugo Nishijima, Takehiro Uda, Vich Yindeedej, Toshiyuki Kawashima, Yuta Tanoue, Takeshi Inoue, Ichiro Kuki, Masataka Fukuoka, Megumi Nukui, Shin Okazaki, Noritsugu Kunihiro, Ryoko Umaba, Kotaro Ishimoto, Hiroshi Uda, Takeo Goto

**Affiliations:** 1 Department of Neurosurgery, Graduate School of Medicine, Osaka Metropolitan University, Osaka 545-8585, Japan; ibericobutan@blue.ocn.ne.jp (S.N.); vichy@tu.ac.th (V.Y.); toshiyuki1986.331.24ser@gmail.com (T.K.); yuta_tanoue_0712@yahoo.co.jp (Y.T.); gotot@omu.ac.jp (T.G.); 2 Department of Pediatric Neurosurgery, Osaka City General Hospital, Osaka 534-0021, Japan; nori9216@omu.ac.jp (N.K.); ryocoumaba@gmail.com (R.U.); noukotaro@gmail.com (K.I.); 3 Division of Neurosurgery, Department of Surgery, Thammasat University Hospital, Faculty of Medicine, Thammasat University, Pathumthani 12120, Thailand; 4 Department of Pediatric Neurology and Neurolinguistics, Osaka City General Hospital, Osaka 534-0021, Japan; takeshiinoue2004@yahoo.co.jp (T.I.); gpkichiro0926@yahoo.co.jp (I.K.); takataka_0730@yahoo.co.jp (M.F.); m.nukui.0730@gmail.com (M.N.); sokazaki2009@gmail.com (S.O.); 5 Department of Pediatrics, Children’s Hospital of Michigan, Detroit Medical Center, Wayne State University, Detroit, MI 48201, USA; hm9480@wayne.edu

**Keywords:** coherence analysis, interhemispheric coherence, electroencephalography, generalized epilepsy, corpus callosum, functional connectivity

## Abstract

**Background:** Interhemispheric coherence, a coherence value between symmetrically opposite electroencephalography (EEG) electrodes, can be considered as a representation of connectivity through commissural fibers. In general, these commissural fibers are the major pathway of communication between hemispheres. However, in patients with drug-resistant generalized epilepsy (GE), these fibers also play an important role in propagating seizure activities to the contralateral hemisphere. The differences in interhemispheric coherence between epilepsy patients and patients without epilepsy (non-E) remain poorly understood. This study compared interhemispheric coherence values between these groups and discussed the potential usage of coherence analysis in the field of epilepsy. **Methods:** We retrospectively collected EEG data from patients with severe non-lesional GE over 20 years old who underwent corpus callosotomy. To compare interhemispheric coherence, EEG data from 10 non-E patients were prepared. In each patient, EEG data during non-rapid eye movement (NREM) sleep were collected. Interhemispheric coherence in eight pairs of electrodes in five frequency bands was calculated. Interhemispheric coherence values were compared between GE and non-E groups. **Results:** In each frequency band and electrode pair, interhemispheric coherence values of P3-P4 in delta, C3-C4 in theta, C3-C4 in alpha, F3-F4 and C3-C4 in beta, and C3-C4 and P3-P4 in gamma frequency band were significantly lower for GE than for non-E. The overall interhemispheric coherence value was significantly lower for GE than for non-E. **Conclusions:** Interhemispheric coherence values were lower for severe GE than for non-E in adults during NREM sleep.

## 1. Introduction

Connectivity analyses have been applied for assessing functional networks in higher brain function, but few studies have been undertaken in the clinical field of epilepsy [1]. Among the various methods of connectivity analysis, coherence analysis offers a quantitative value representing the correlation of amplitude between pairs of electrodes [2,3].

Interhemispheric coherence is a coherence value between symmetrically opposite electroencephalography (EEG) electrodes and can be considered to represent connectivity through commissural fibers, including the corpus callosum, anterior commissure, and posterior commissure. In general, these commissural fibers represent the major pathway of communication between both hemispheres. However, in patients with drug-resistant generalized epilepsy (GE), the same fibers play an important role in propagating seizure activities to the contralateral hemisphere [4].

We recently reported that interhemispheric coherence values decreased following corpus callosotomy for refractory epilepsy [5]. We also demonstrated that preoperative interhemispheric coherence values were associated with seizure outcomes after corpus callosotomy [6]. However, differences in interhemispheric coherence between epilepsy patients and regular population are not yet understood.

We hypothesized that interhemispheric coherence would differ between adult patients with severe drug-resistant GE and patients without epilepsy (non-E). In this study, we compared interhemispheric coherence values between these groups and discussed the potential usage of coherence analysis in the field of epilepsy.

## 2. Materials and Methods

### 2.1. Patients

We retrospectively collected data from patients with medically intractable, non-lesional severe GE over 20 years old who underwent corpus callosotomy between 2014 and 2023 at Osaka Metropolitan University Hospital and Osaka City General Hospital. All patients had a high seizure frequency and a substantial risk of falls and injury. The diagnosis was established by integrating detailed clinical history, seizure semiology, imaging findings with magnetic resonance imaging and positron emission tomography, and findings from prolonged preoperative video- EEG monitoring. In EEG, the inclusion required the presence of bilateral and synchronous spike–wave discharges or generalized epileptiform activity on preoperative EEG, without the evidence of a consistent unilateral focal onset. No sedative agents were used during video EEG monitoring, Patients for whom the focality of seizures had been revealed after corpus callosotomy were excluded. The age at surgery, sex, seizure type, frequency of seizure, amount of antiseizure medicine, duration of epilepsy, and neurocognitive status were collected. The degree of neurocognitive decline before surgery was divided into four categories based on daily life activity: none, mild, moderate, and severe. Patients fully capable of daily activities without assistance were categorized to the “none” group. Those with minor limitations were placed in the “mild” group, patients requiring some assistances were categorized to the “moderate” group, and those needing help with most daily activities were classified to the “severe” group. To compare interhemispheric coherence, EEG data from ten non-E patients with similar age distribution were collected as controls. These non-E patients had been referred to our institution on suspicion of GE due to symptoms such as loss of consciousness, abnormal behavior, or psychiatric symptoms. They were subsequently confirmed to show no abnormalities on either EEG and magnetic resonance imaging and to have no psychiatric disorders.

The present study was approved by the institutional review boards at Osaka Metropolitan University Hospital (No. 2023-124) and Osaka City General Hospital (No. 2401145).

### 2.2. EEG Data Recording and Acquisition

EEG data were recorded with 19 scalp electrodes placed according to the international 10–20 system. The Neurofax EEG system was used (Nihon Kohden Co., Tokyo, Japan) and the sampling rate was 200, 500, or 1000 Hz depending on clinical recording conditions. All sampling rates sufficiently covered the analyzed frequency range (<80 Hz) described below. In each patient, EEG data during non-rapid eye movement (NREM) sleep were collected. The reasons for selecting NREM sleep for the analysis are as follows. First, cognitive impairment in patients makes it difficult to obtain stable resting-state awake EEG recordings free from movement-related electromyographic artifacts. Second, EEG during rapid eye movement sleep is frequently contaminated by eye movements and body motion artifacts, making it less suitable for reliable coherence analysis. In contrast, spontaneous movements are reduced during NREM sleep, allowing the acquisition of EEG data with fewer artifacts. Third, in our previous studies, interhemispheric coherence was analyzed using EEG recordings obtained during NREM sleep. Sleep stages were visually scored by two physicians experienced in clinical neurophysiology (TU and SN). In the present study, subsequent analyses were conducted using EEG segments without epileptic discharges to eliminate their potential influence on coherence values. Epileptic discharges were defined as generalized spike-and-wave discharges, polyspikes, and spike-and-wave complexes, and any EEG segments containing these patterns were manually excluded from the analysis. In addition, to minimize the effects of artifacts on coherence values, segments containing eye movements, electromyographic activity, body movements, or electrode noise were manually removed. Particular care was taken with high frequency activity in frontal and temporal electrodes, which may influence gamma-band coherence. Finally, the remaining data were concatenated to generate consecutive artifact-free segments with a total duration of 60 s [5,6].

### 2.3. Interhemispheric Coherence Values

Interhemispheric coherence values were calculated using the Data Editor function in the EMSE software version 6.5 (Cortech Solutions, Wilmington, NC, USA). In this study, a linked-ears reference was used, and no additional re-referencing was performed prior to coherence calculation. For spectral analysis, the window duration was set to 1 s, with 50% overlap between consecutive windows. Coherence values for each frequency band were calculated as the average of coherence values across all frequency components within the respective band. Coherence values range from 0 to 100, with a coherence value of 100, indicating that two EEG waveforms are identical in amplitude. On the other hand, a coherence value of 0 indicates that two EEG waveforms are completely different in amplitude.

**Figure 1:**
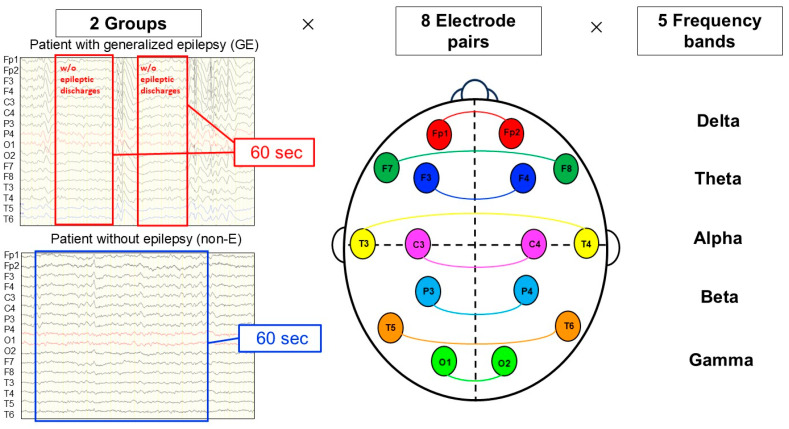
Flow of coherence analysis. Segments without any visible artifacts and epileptic discharges were collected and combined as consecutive data of 60 s in total for each patient. Interhemispheric coherence values from the eight pairs of symmetrically opposite scalp electrodes (Fp1-Fp2, F3-F4, F7-F8, C3-C4, T3-T4, T5-T6, P3-P4, and O1-O2) in 5 frequency bands delta [0.1–4 Hz], theta [4–8 Hz], alpha [8–13 Hz], beta [13–30 Hz], and gamma [30–80 Hz] were calculated.

Interhemispheric coherence values from the eight pairs of symmetrically opposite scalp electrodes (Fp1-Fp2, F3-F4, F7-F8, C3-C4, T3-T4, T5-T6, P3-P4, and O1-O2) in 5 frequency bands delta [0.1–4 Hz], theta [4–8 Hz], alpha [8–13 Hz], beta [13–30 Hz], and gamma [30–80 Hz] were calculated. The 40 interhemispheric coherence values were obtained for each patient [(8 electrode pairs) × (5 frequency bands)] [5]. (Figure 1)

### 2.4. Evaluations

We evaluated the significance of median differences in interhemispheric coherence for each frequency band and each electrode pair using the Wilcoxon rank-sum test, with the adjustment for multiple comparisons through the Bonferroni correction. As described above, interhemispheric coherence was calculated for eight electrode pairs and five frequency bands per participant, resulting in a total of 40 comparisons. Therefore, a corrected significance threshold of *p* = 0.05/40 = 0.00125 was applied. Next, we performed statistical analyses using a linear mixed-effects model to examine whether interhemispheric coherence differed between the GE and non-E group, independent of frequency band and electrode pair. Interhemispheric coherence was modeled as the outcome variable, with group, frequency band, and electrode pair included as fixed effects; sex and age as covariates; and patient as a random effect. Fixed effects and their statistical significance were evaluated using point estimates, 95% confidence intervals, and *p* values; t values, standard errors, and degrees of freedom were also reported.

Statistical analyses were carried out using the JMP software (version 14.0; SAS Institute, Cary, NC, USA). The differences between the two groups were analyzed using the Wilcoxon rank-sum test. To compare demographic data between both groups, values of *p* < 0.05 were considered statistically significant. To compare each electrode pair and frequency band in both groups, values of corrected *p* < 0.05 (=0.00125) were considered statistically significant. All mixed-effects model analyses were performed using the Statistics and Machine Learning Toolbox in MATLAB R2025a (MathWorks, Natick, MA, USA) and values of *p* < 0.05 were considered statistically significant.

## 3. Results

### 3.1. Patient Demographics

Nine patients with GE and ten patients with non-E were included in this study. In GE, mean age at EEG acquisition was 29.4 years (range, 20–43 years) and the patients comprised seven men and two women. Median duration of epilepsy was 22 years (range, 14–43 years). Seizure types were epileptic spasm and tonic–clonic seizure in two patients, tonic–clonic seizures in five patients, epileptic spasm and tonic seizure in one patient each. Neurocognitive decline was classed as “none” in one patient, “mild” in two patients, “moderate” in one, and “severe” in five patients. The patients had seizures occurring daily to monthly and were treated with multiple antiseizure medications. Patient characteristics in GE are presented in Table 1.

In non-E, mean age was 33.8 years (range, 22–45 years) and patients comprised two men and eight women. No significant difference was observed in terms of age between patients with GE and non-E (*p* = 0.1785). Reasons for EEG evaluation were loss of consciousness in two patients, abnormal behavior in four and other psychiatric symptoms in four patiens. Patient characteristics in non-E are presented in Table 2.

### 3.2. Comparing Interhemispheric Coherence in Each Frequency Band and Electrode Pairs 

**Figure 2:**
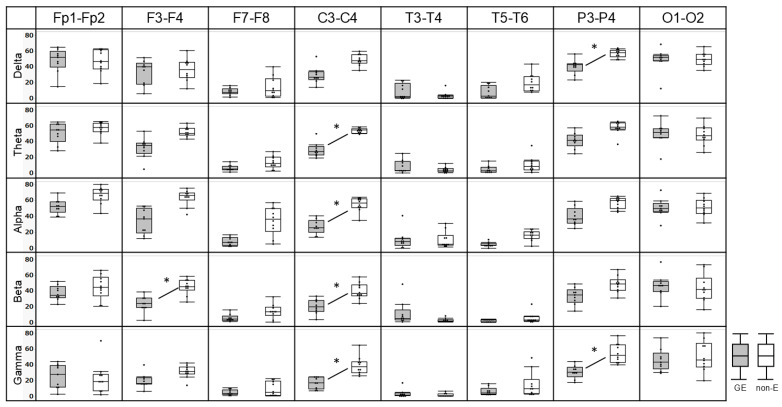
Scatter and box-and-whisker plots comparing interhemispheric coherence of GE (gray) and non-E (white). Comparing interhemispheric coherence in each frequency bands electrode pair, interhemispheric coherence values of P3-P4 in delta, C3-C4 in theta, C3-C4 in alpha, F3-F4 and C3-C4 in beta, and C3-C4 and P3-P4 in gamma frequency band (asterisk, *) were significantly lower for GE than for non-E (*p* = 0.0009, 0.0004, 0.0005, 0.0009, 0.0009, and 0.0003, respectively).

In each frequency band and electrode pair, interhemispheric coherence values of P3-P4 in delta, C3-C4 in theta, C3-C4 in alpha, F3-F4 and C3-C4 in beta, and C3-C4 and P3-P4 in gamma frequency band were significantly lower for GE than for non-E after Bonferroni multiple comparison correction (*p* = 0.0009, 0.0004, 0.0005, 0.0009, 0.0009, and 0.0003, respectively) (Figure 2).

### 3.3. Comparing Interhemispheric Coherence in All Frequency Bands and Electrode Pairs

Median overall interhemispheric coherence value for GE was 23.3 (interquartile range [IQR] 6.8–40.9) and 39.2 (IQR 13.1–54.8) for non-E.

Interhemispheric coherence was significantly lower in the GE group than in the non-E group, regardless of the frequency band or electrode pair (estimate = −7.79, 95% CI −9.88–−5.77, *p* < 0.001). Age was not significantly associated with interhemispheric coherence (*p* = 0.19); however, sex was a significant covariate, and coherence was lower in males (estimate = −3.88, *p* < 0.001). Detailed statistical results are provided in Table 3.

## 4. Discussion

### 4.1. The Summary of the Present Study

In each frequency band and electric pair, interhemispheric coherence values of P3-P4 in delta, C3-C4 in theta, C3-C4 in alpha, F3-F4 and C3-C4 in beta, and C3-C4 and P3-P4 in gamma frequency band were significantly lower for GE than for non-E. The overall interhemispheric coherence value was significantly lower for GE than for non-E during the period without epileptic discharges in NERM sleep. Coherence has previously been used in epilepsy research, particularly to investigate functional connectivity and its association with cognitive impairment in patients with epilepsy. However, the novelty of the present study lies in the direct comparison of interhemispheric coherence between adults with GE and non-E using NREM sleep EEG data without epileptic discharges.

### 4.2. Possible Reasons for Lower Interhemispheric Coherence in GE

One possible reason for lower interhemispheric coherence in GE than in non-E might be a difference in the roles of the corpus callosum. In non-E, the corpus callosum plays crucial roles in mediating various information from one hemisphere to the other [7]. In GE, the corpus callosum plays another role in propagating epileptic activity [8,9]. This role might affect interhemispheric coherence values. Previous studies have reported pathophysiological abnormalities of the corpus callosum in GE, such as a decreased volume of the corpus callosum and alterations in the white matter, including decreased axonal integrity and disrupted structural alignment [10,11]. These abnormal changes might lead to the development of abnormal functional network via the corpus callosum and might display some relations related to seizure propagation [12,13]. The lower interhemispheric coherence in GE compared with non-E might be the result of impaired functional connectivity via the corpus callosum.

Several studies have reported reduced coherence in patients with neurocognitive dysfunction, which could be related to disrupted functional connectivity [14]. Such disruptions in functional connectivity can predispose patients to the formation of aberrant neural networks, potentially facilitating epileptogenesis [15]. In addition, recurrent epileptic activity might disrupt functional connectivity and further exacerbate neurocognitive dysfunction [16]. In fact, GE in this study mostly had neurocognitive dysfunction, and this interaction could impair functional connectivity, which might have affected the fact that GE showed lower interhemispheric coherence than non-E.

### 4.3. Influence of Sex and Age as Covariates in the Mixed-Effects Model

In the mixed-effects model analysis, age was not significantly associated with interhemispheric coherence, whereas sex emerged as a statistically significant covariate, with lower coherence observed in male patients. Although this finding reached statistical significance, its physiological and clinical relevance remains unclear. Given the observational nature of the present study and the absence of a priori hypotheses regarding sex-dependent differences in interhemispheric coherence, this result should be interpreted with caution. It is possible that the observed association reflects sample-specific characteristics or residual confounding related to the limited number of clinically acquired cases rather than a true underlying biological effect. Future studies with larger sample sizes and more balanced sex distributions will be necessary to clarify whether sex exerts a meaningful influence on interhemispheric coherence.

### 4.4. Limitations

This study has several limitations. First, it was a retrospective, preliminary investigation of a highly selected cohort of adults with drug-resistant severe generalized epilepsy who were candidates for corpus callosotomy, resulting in a small sample size and limited statistical power, which may explain the lack of significance in some frequency bands or electrode pairs. Second, the control group consisted of patients with paroxysmal symptoms rather than healthy volunteers; thus, unrecognized psychiatric or cognitive factors may have affected interhemispheric coherence, limiting the generalizability of the findings. Third, cognitive impairment was common in the generalized epilepsy group, and reduced coherence may therefore reflect diffuse cerebral dysfunction rather than epilepsy itself, precluding causal inference. Fourth, stratified analyses by seizure type and quantitative comparisons among NREM sleep stages were not feasible because of the limited sample size. Finally, quantitative MRI assessments of the corpus callosum and comprehensive cognitive evaluations were not included. Future large-scale prospective studies incorporating healthy controls, neuropsychological testing, and structural neuroimaging are needed to clarify the clinical and pathophysiological significance of altered interhemispheric functional connectivity in generalized epilepsy.

## 5. Conclusions

Interhemispheric coherence values were significantly lower for severe GE than for non-E in adults during the period without epileptic discharges in NERM sleep.

## Figures and Tables

**Table 1:** Characteristics of patients with generalized epilepsy.

Patient	Age (Years)	Sex	Seizure Type	Duration of Epilepsy (Years)	Neuro-Cognitive Decline	Seizure Frequency	Antiseizure Medications (mg/Day)
1	20	M	ES/tonic–clonic	19	Severe	Daily	CLB5, TPM30, VPA900
2	22	M	ES/tonic–clonic	22	Mild	Weekly	LCM400, VPA900
3	27	F	tonic–clonic	18	None	Weekly	CLB15, LEV2000, PHT200, VPA800
4	27	M	ES	14	Severe	Daily	CZP2, PHT150, LEV1500, LTG100, VPA800
5	30	M	tonic–clonic	14	Moderate	Weekly	CLB10, LEV3000, PER2, PHT200, TPM200, VPA1200, ZSM300
6	31	M	tonic–clonic	28	Mild	Weekly	CLB12, PER4, VPA600
7	32	M	tonic–clonic	31	Severe	Daily	LEV3000, PER12, PHT200, TPM200, VPA1200
8	33	M	tonic–clonic	32	Severe	Monthly	CLB5, PER4, VPA1200
9	43	F	tonic	43	Severe	Daily	CNP12, VGB400, ZSM100

ES: epileptic spasm, F: female, M: male, CLB: clobazam, CZP: clonazepam, LEV: levetiracetam, LTG: lamotrigine, PER: perampanel, PHT: phenytoin, TPM: topiramate, VGB: vigabatrin, VPA: valproic acid, ZSM: zonisamide.

**Table 2:** Characteristics of patients without epilepsy.

Patient	Age (Years)	Sex	Reason for EEG Evaluation
1	22	F	Syncope
2	27	M	Abnormal behavior
3	28	F	Psychiatric symptoms
4	31	F	Syncope
5	34	F	Abnormal behavior
6	34	M	Psychiatric symptoms
7	38	F	Psychiatric symptoms
8	39	F	Abnormal behavior
9	39	F	Psychiatric symptoms
10	45	F	Abnormal behavior

F: female, M: male.

**Table 3:** Mixed-effects model for interhemispheric coherence value.

		Mixed-Effects Model Coefficient			*p* Value	SE	t Value	DF
		Estimate	95% CI (Lower)	95% CI (Upper)				
(Intercept)		51.64011827	45.78652014	57.49371641	1.00393 × 10^−56^	2.981732522	17.31882987	745
Group	GE	−7.791191306	−9.883001424	−5.699381188	6.80606 × 10^−13^	1.06553578	−7.311994069	745
	non-E	(Control)						
Frequency Band	Beta	−9.825723684	−12.51369599	−7.137751381	1.73519 × 10^−12^	1.369211593	−7.176190832	745
	Delta	−5.236447368	−7.924419671	−2.548475066	0.000142047	1.369211593	−3.824425234	745
	Gamma	−12.70638599	−15.39435894	−10.01841303	1.80561 × 10^−19^	1.369211927	−9.28007253	745
	Theta	−3.940723684	−6.628695987	−1.252751381	0.004115343	1.369211593	−2.878096931	745
	Alpha	(Control)						
Electrode Pair	F3-F4	0.916105263	−2.483940643	4.316151169	0.596997522	1.731930894	0.528950241	745
	F7-F8	−24.32884211	−27.72888801	−20.9287962	6.18772 × 10^−40^	1.731930894	−14.04723606	745
	Fp1-Fp2	8.685263158	5.285217252	12.08530906	6.64165 × 10^−7^	1.731930894	5.014786208	745
	O1-O2	12.00020347	8.600156244	15.4002507	9.19114 × 10^−12^	1.731931568	6.928797705	745
	P3-P4	9.948842105	6.548796199	13.34888801	1.34428 × 10^−8^	1.731930894	5.744364364	745
	T3-T4	−29.97831579	−33.3783617	−26.57826988	1.1313 × 10^−56^	1.731930894	−17.30918705	745
	T5-T6	−26.95926316	−30.35930906	−23.55921725	1.6397 × 10^−47^	1.731930894	−15.56601551	745
	C3-C4	(Control)						
Age		−0.094664976	−0.237177639	0.047847687	0.192623542	0.07259375	−1.30403754	745
Sex	Male	−3.878808697	−6.112842745	−1.64477465	0.000688342	1.137982454	−3.408496048	745
	Female	(Control)						

CI: confidence interval, SE: standard error, DF: degrees of freedom.

## Data Availability

The data presented in this study are available on request from the corresponding author. The data are not publicly available due to privacy or ethical restrictions.

## Abbreviations

The following abbreviations were used in this manuscript:

GE	generalized epilepsy
non-E	non-epilepsy
EEG	electroencephalography
NREM	non-rapid eye movement
